# Fabrication of Aliphatic Water-Soluble Polyurethane Composites with Silane Treated CaCO_3_

**DOI:** 10.3390/polym12040747

**Published:** 2020-03-29

**Authors:** Eyob Wondu, Zelalem Chernet Lule, Jooheon Kim

**Affiliations:** School of Chemical Engineering and Materials Science, Chung-Ang University, Seoul 156-756, Korea; wendueyoba@gmail.com (E.W.); zochernet@gmail.com (Z.C.L.)

**Keywords:** water-soluble polyurethane, hydroxyl-terminated polybutadiene, water solubility, CaCO_3_, composites

## Abstract

In the present study, composites of water-soluble polyurethane/calcium carbonate (CaCO_3_) were prepared from a soft segment of hydroxyl-terminated polybutadiene (HTPB) and polyethylene glycol (PEG, average molecular weight = 4000) with aliphatic diisocyanates. The functionality of CaCO_3_ particles was modified using aminopropyltriethoxysilane (APTES), and was confirmed by Fourier-transform infrared spectroscopy (FTIR). The solubility, hydrophilic properties, and chemical structures of the composites were analyzed by water-solubility tests, contact angle measurements, and FTIR, respectively, and the successful production of the hydrophilic water-soluble polyurethane (WSPU) structure was demonstrated. The adhesion of surface-modified CaCO_3_ particles to the WSPU matrix and the thermal degradation properties of the neat WSPU and WSPU/CaCO_3_ composites were studied using field emission scanning electron microscopy (FE-SEM) and thermogravimetric analysis (TGA). The results demonstrated good adhesion of the surface-modified CaCO_3_ particles along with an improved thermal degradation temperature with the addition of CaCO_3_ particles to the WSPU matrix.

## 1. Introduction

Polyurethane (PU) composites can be used in adhesive industries, synthetic leathers, construction industries, automotive industries, coatings, etc. In particular, water-soluble polyurethane (WSPU) is highly desired due to the need for highly flexible but tough polymers with enhanced adhesion, resistance to abrasion, and resistance to chemicals (including solvents and pollutants) [1,2,3,4]. In comparison to WSPU, conventional PU is a pollutant and is toxic to the environment [5,6]. Moreover, although the neat WSPU has the practical disadvantages of poor thermal, mechanical, and adhesion properties in the ambient environment [4,7,8,9], improvements in these properties are provided by the polymer composites.

Present-day research efforts in academia and industry alike are primarily focused on the development of environmentally benign polymer composites [8,10,11,12,13,14]. Hence, the development of WSPU composites by the incorporation of various fillers to enhance the thermo-mechanical and adhesion properties while causing low environmental harm and pollution is essential to the present-day research areas in PU industries. Among the various kinds of fillers, calcium carbonate (CaCO_3_) is particularly attractive due to its high commercial availability, along with its ability to significantly enhance polymer properties [1,9,10]. WSPU/CaCO_3_ composites can be applied in coating industries and automotive and adhesive industries, as CaCO_3_ enhances the properties of the WSPU matrix [9]. X. GaO et al. have fabricated WSPU/CaCO3 composites using polypropylene glycol as a soft segment and TDI as hard segment by treating the surface of CaCO_3_ with oxalic acid. They found that incorporating CaCO3 did not affect WSPU/CaCO3 thermal stability [9]. Although in-situ polymerization is the preferred method to produce polymer composites with high thermal stability, the use of CaCO_3_ as a filler in WSPU composites via this method is very rare. In addition, the large specific surface area of the CaCO_3_ particles promotes the formation of aggregates during the preparation of composites. This leads to the formation of an incompatible surface and difficulties in forming a uniform distribution of the filler in the matrix [4,15,16]. Therefore, the surface of the CaCO_3_ particles must be modified to enable their use in the formation of a uniform WSPU matrix with good surface compatibility. In present-day research in the field of composite materials, aminopropyltriethoxysilane (APTES) is a well-known surface-modifying reagent for the treatment of various fillers [3,8,12,15,17].

The various raw materials used in the production of WSPUs include the diisocyanates, chain extenders, and emulsifiers, which generate the hard segments of the polymer chain, and the polyols, which constitute the soft segments. The types of diisocyanates that are available include aromatic diisocyanates, which are susceptible to UV degradation and are used where this is not a problem, and aliphatic diisocyanates, which are resistant to UV degradation and yellowing [1,3,5,7,9,14,16,17,18,19,20,21,22,23,24,25,26,27]. The aliphatic diisocyanates include 4,4’-methylenebis(cyclohexyl isocyanate) (HMDI), isophorone diisocyanate (IPDI), tetramethyl xylene diisocyanate (TMXDI), and hexamethylene diisocyanate (HDI); the aromatic types are comprised of methylenebis(phenyl isocyanate) (MDI) and toluene diisocyanate (TDI) [28]. In addition, X. Wang. et al. reported the fabrication of graphene-reinforced WSPU composites, in which they treated the surface of graphene oxide using APTES and demonstrated that the addition of graphene oxide enhanced the WSPU’s thermal stability [20].

In the present study, the aliphatic diisocyanate IPDI was selected along with dimethylolpropionic acid (DMPA) as an emulsifier and butane diol (BD) as a chain extender. Triethylamine (TEA) was used to neutralize the carboxyl group in DMPA, and the soft segment consisted of hydroxyl-terminated polybutadiene (HTPB)-polyethylene glycol (PEG, average molecular weight = 4000). The presence of the same functional groups (hydroxyl groups) on both HTPB [OH-(CH2-CH=CH-CH2)-)_n_OH] and PEG [H(-O-CH2-CH2-)_n_OH] made the two soft segments compatible to each other [29]. Moreover, PEG was selected due to its hydrophilic property, while the hydrophobic HTPB was chosen to preserve the properties of the final WSPU [30]. The surface of CaCO3 was modified by APTES, and the polymer composite was fabricated via the in-situ polymerization method. Since there has not yet been any work on the HTPB-PEG-based WSPU composite, to the best of the present authors’ knowledge, the present work can promote further investigations in this area.

## 2. Materials and Methods

### 2.1. Materials

Isophorone diisocyanate (IPDI, 98%), dibutyltin dilaurate (DBTDL, 99%), dimethylolpropionic acid (DMPA, 98%), triethylamine (TEA, ≥ 99%,), aminopropyltriethoxysilane (APTES), and 1,4-butane diol (BD, 99%,) were obtained from Sigma-Aldrich (St. Louis, MO, USA). Polyethylene glycol 4000 (PEG4000), ethanol (>94.5%), and tetrahydrofuran (THF, >99%) were supplied by Dae-Jung Chemical and Metal Co. Ltd. (Seoul, Korea). Hydroxyl-terminated polybutadiene (HTPB) and calcium carbonate (CaCO_3_, average size 10 µm) were kindly supplied by the South Korea Agency for Defense Development (ADD). All chemicals were used as received.

### 2.2. Methods

#### 2.2.1. Surface Modification of CaCO_3_

**Figure 1:**
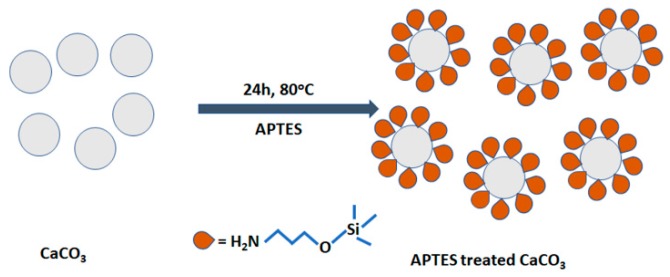
Scheme showing the aminopropyltriethoxysilane (APTES) treatment of calcium carbonate (CaCO_3_) particles.

The following procedures were used in modifying the surface of the CaCO_3_ particles. The CaCO_3_ particles were dried in an oven at 80 °C for about 24 h, then subjected to surface modification for about 6 h at 80 °C using APTES (5 wt.% of CaCO_3_) in ethanol as a solvent. The surface-modified CaCO_3_ particles were then washed two times by filtration with water and dried in an oven for 24 h. The APTES treatment of CaCO_3_ particles is presented in Figure 1.

#### 2.2.2. Synthesis of Water-Soluble Polyurethane/CaCO_3_ Composites

**Figure 2:**
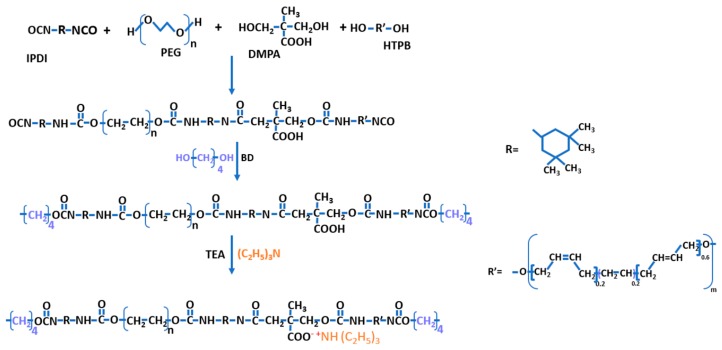
The structures of each chemical, the intermediates involved, and the synthetic route of water-soluble polyurethane (WSPU).

The preparation of the water-soluble polyurethane/calcium carbonate (WSPU/CaCO_3_) composite was performed in a three-necked round-bottomed flask equipped with a mechanical stirrer, condenser, and dropping funnel, under a dry nitrogen atmosphere in a constant temperature oil bath. The PEG and HTPB polyols were added to THF (30 mL) in a 1:1 ratio and mixed with the proper amount of CaCO_3_ at 80 °C for 2h. After reducing the temperature to 60 °C, 10 wt.% of DMPA (with respect to HTPB and PEG) was added to the reaction vessel, followed by the simultaneous addition of 1:3 HTPB:IPDI and three drops of DBTDL. The reaction was allowed to proceed for 2 h prior to the addition of BD as a chain extender and stirring for an additional 1 h at the same temperature. Finally, the temperature was reduced to 50 °C, TEA (neutralizer) was added, and the mixture was stirred for 30 min. The chemical structures each chemical used as raw materials to produce the WSPU; the intermediates involved and the synthetic route of WSPU are presented in Figure 2. The samples were prepared with 10wt.% CaCO_3_ for both pristine and APTES-treated CaCO_3_ (WSPU/10CaCO_3_ and WSPU/10TCaCO_3_, respectively), and 20wt.% CaCO_3_ for both pristine and APTES-treated CaCO_3_ (WSPU/20CaCO_3_, WSPU/20TCaCO_3_ respectively), where T stands for APTES-treated, with respect to the weight of PEG and HTPB.

### 2.3. Characterization

The chemical structures of the WSPU and WSPU/CaCO_3_ composites, and that of the CaCO_3_ after surface modification with APTES, were confirmed by Fourier-transform infrared spectroscopy (FT-IR; Nicolet, is5, Thermo Fisher Scientific, Seoul, Korea). For the samples of CaCO_3_ APTES attachment confirmation, FTIR specimens were prepared by utilization of potassium bromide (KBr) background to make flakes with a 4000–650 frequency range.

The surface morphology and adhesion of the WSPU and WSPU/CaCO_3_ composites were studied using field emission scanning electron microscopy (FE-SEM; Sigma, Carl Zeiss, Oberkochen, Germany) upon fracturing the specimens with liquid nitrogen and coating with platinum under a vacuum to inhibit the accumulation of charge due to the release of electrons during the analysis. FE-SEM was also used to determine the size of the CaCO_3_ particles and to determine whether the APTES was attached to the CaCO_3_ particles using energy dispersive spectroscopy (EDS).

The thermal stabilities of 5 mg samples of the neat WSPU and WSPU/CaCO_3_ were investigated by thermogravimetric analysis (TGA-2050, TA Instruments, New Castle, DE, USA). The samples were heated from 25 to 600 °C with a heating rate of 10 °C/min in a nitrogen atmosphere.

In addition, the water-solubility test analysis was performed by pouring the WSPU/CaCO_3_ into a vial of water and stirring for three hours at room temperature. The hydrophobic properties of the neat WSPU and the WSPU/CaCO_3_ composites were analyzed at room temperature with 5 μL of water by the sessile drop method using a drop shape analyzer (DSA100, KRÜSS GmbH, Hamburg, Germany). The contact angles were measured after all the samples were made flat using a hot press at 65°C for 20 min, except for the neat WSPU which formed a film. The average values of the contact angles were calculated after five repeat measurements of a single specimen.

## 3. Results and Discussion

Solution process is a process of producing polymers using a solvent that can dissolve all raw materials involved in the production process. Various solvents, for instance, acetone, dichloromethane, methanol, dimethylfuran, and tetrahydrofuran, were tested for their applicability to the solution process, with the following outcomes: acetone was unable to dissolve the HTPB, dichloromethane and methanol were unable to dissolve the DMPA, dimethylfuran sparingly dissolved the DMPA, while tetrahydrofuran (THF) was finally selected for its ability to dissolve all the raw materials. The solvent was used in order to reduce the development of viscosity during polymer fabrication.

**Figure 3:**
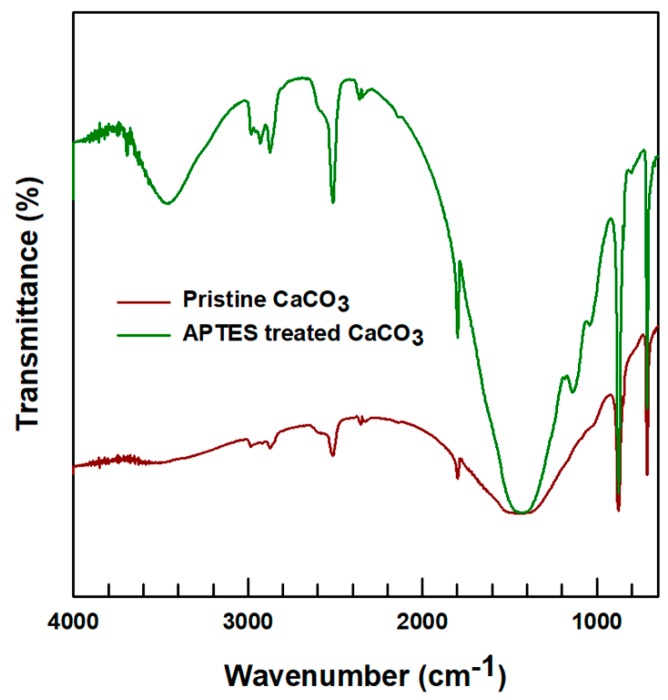
FTIR spectra of the pristine and APTES treated CaCO_3_.

The FTIR spectra of the pristine and surface-modified CaCO_3_ are presented in Figure 3. The peaks at wavenumbers of 2900 and 2860 cm^−1^, found for both, were due to the –CH_2_ stretching mode, and those at around 1421 and 712 cm^−1^ were due to the asymmetric stretching and in plane bending of carbonates. Moreover, a wide peak due to the -NH_2_ (amine group) originating from APTES was observed on the APTES-treated CaCO_3_ spectrum at a wavenumber of 3500-3300 cm^−1^, thus confirming the attachment of APTES to the CaCO_3_ particles. In addition, the peaks around 1141 and 800 cm^−1^ of the APTES-treated CaCO_3_ corresponded to Si–O–C and Si–O–Si, respectively, which indicated that the APTES was attached to the CaCO_3_ particles.

**Figure 4:**
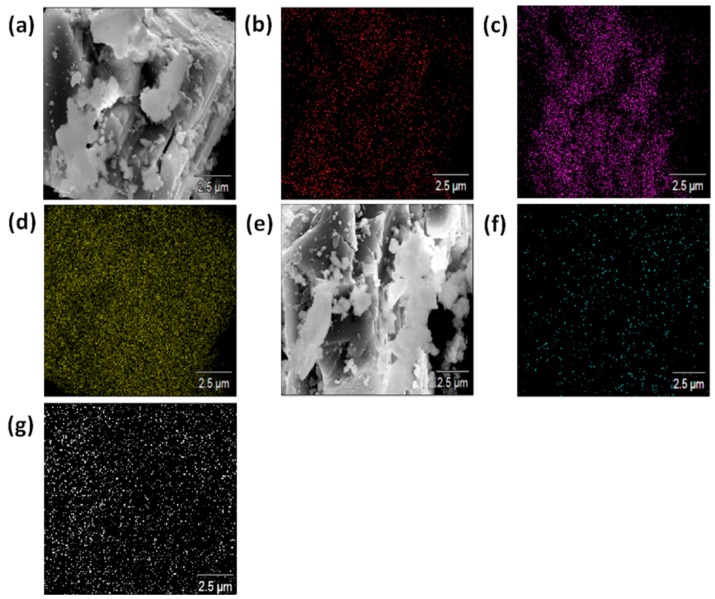
SEM and energy dispersive spectroscopy (EDS) analyzes of the pristine (**a**–**d**) and APTES treated (e-g) CaCO_3_: (**a**) SEM image, (**b**) carbon mapping, (**c**) oxygen mapping, (**d**) calcium mapping, (**e**) SEM image, (**f**) nitrogen mapping, and (**g**) silicon mapping.

The morphologies of the pristine and APTES-treated CaCO_3_ particles was studied by using FE-SEM and EDS analysis, as shown in Figure 4. The FE-SEM results of pristine CaCO_3_, Figure 4a, revealed the presence of some surface impurities, including Mg, which appears predominantly next to Ca, C, and O atoms of CaCO_3_. The EDS analysis was performed to confirm the attachment of APTES to the CaCO_3_ particles. First, the chemical composition of the CaCO_3_ particles (Figure 4a) was confirmed by the carbon EDS mapping (Figure 4b), the oxygen EDS mapping (Figure 4c), and the calcium EDS mapping (Figure 4d). Then, the attachment of APTES to the CaCO_3_ (Figure 4e) was confirmed by the nitrogen EDS mapping (Figure 4f) and the silicon EDS mapping (Figure 4g). Neither nitrogen nor silicon was detected on the pristine CaCO_3_.

**Figure 5:**
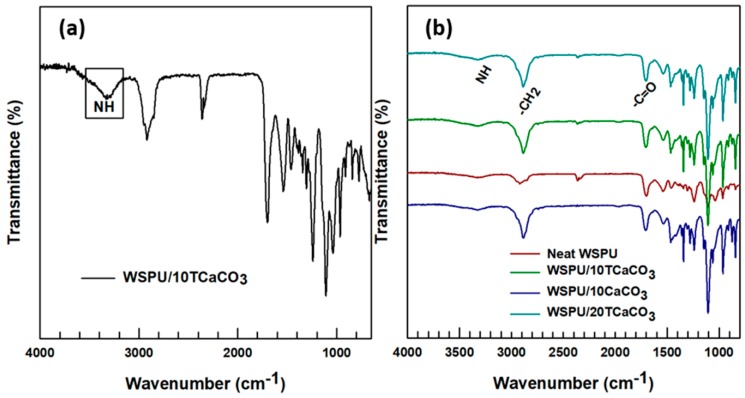
FTIR spectra of (**a**) the WSPU/10TCaCO_3_ composite; (**b**) the neat WSPU and various WSPU/CaCO_3_ composites on the same chart.

The FTIR spectra of the WSPU and WSPU/CaCO_3_ are presented in Figure 5. The peak at around 3250−3450 cm^−1^ in Figure 5a was attributed to the NH- stretching mode, thus demonstrating the appearance of the PU structure due to the urethane linkage between the polyol and diisocyanate (HTPB-PEG and IPDI). The sharp peak at around a wavenumber of 1726 cm^−1^ confirmed the appearance of the urethane carbonyl groups (C=O) derived from the emulsifier. This group allowed the PU to be water-soluble, which was confirmed in the water-solubility test section. The peaks at around 1311 cm^−1^ were due to the twisting mode of −CH_2_, while that at around 1360 cm^−1^ corresponded to the methylene groups wagging vibrations [3,12,18]. The peaks in the region 2800-3000 cm^−1^ were due to the asymmetric and symmetric stretching modes of the –CH_2_ groups. In the WSPU/CaCO_3_ composites, the addition of CaCO_3_ to the WSPU had no significant effect on the characteristic absorbance peaks, and its influence upon the chemical structure was not visible even for composites containing 20% CaCO_3_.

**Figure 6:**
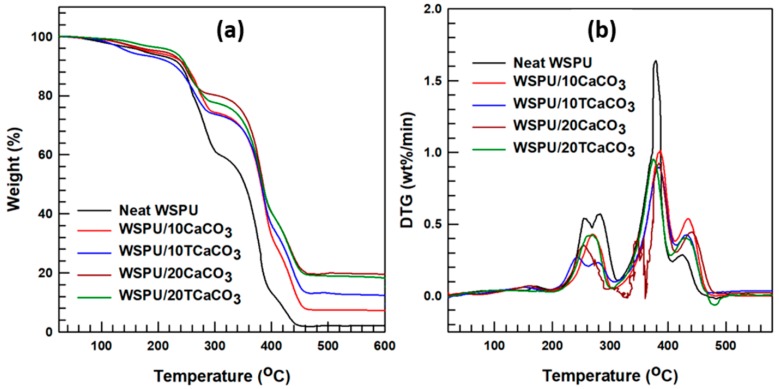
Thermal degradation properties of the WSPU and WSPU/CaCO_3_ composites: (**a**) thermogravimetric analysis (TGA) and (**b**) derivative TGA (DTGA).

The TGA thermograms of the neat WSPU and the WSPU/ CaCO_3_ composites presented in Figure 6 all revealed a closely similar appearance and decomposition pattern. The TGA analysis provided a measure of sample degradation as a function of temperature change. In Figure 6a, the first decomposition peak begins to appear at around 130 °C due to the removal of moisture from the sample specimen. While the initial decomposition occurred between 130 and 230 °C, the second decomposition peak for the WSPU/CaCO_3_ composites appeared at 285 °C, at which stage 25% of the composite was degraded. For the neat WSPU, the second decomposition curve extended to 300 °C, at which stage 40% of the polymer was degraded. While the initial decomposition curves of both the neat WSPU and the WSPU/CaCO_3_ composites were fairly similar in pattern, the second decomposition curve was distinct for the neat WSPU. In detail, the 50 wt.% decomposition (D_1/2_) of the WSPU/CaCO_3_ composite (385 °C) was greater than that of the neat WSPU (350 °C). However, the final decomposition temperature of both the neat WSPU and WSPU/CaCO_3_ composites was 460 °C, with no further weight loss being observed with further increase in temperature above this point. The final residues differed according to the weight percentage of CaCO_3_ used, such that the residue content increased with the increased weight percentage of CaCO_3_. However, for the final residue of WSPU/10TCaCO_3_ as compared to same amount of pristine CaCO_3_ containing WSPU, i.e., WSPU/10CaCO_3_, the amount remaining after degradation was slightly higher, which may have been due to the surface modification that led all the CaCO_3_ to be attached to the matrix.

The derivative TGA (DTGA) plots of the neat WSPU and WSPU/CaCO_3_ composites are presented in Figure 6b. The maximum derivative temperature of the neat WSPU was 377 °C, and that of the WSPU/CaCO_3_ composite was 385 °C, which shows a slightly similar range. Figure 6b of neat WSPU DTGA depicts that the height of the maximum derivative temperature was higher than the others, indicating that the thermal degradation rate of neat WSPU is higher than that of the composites. Therefore, it can be deduced that the neat WSPU degraded faster than the WSPU/CaCO_3_ composites. Thus, in contrast to the work of X. GaO et al. [9], the addition of CaCO_3_ into the WSPU provided more thermal stability to the matrix.

**Figure 7:**
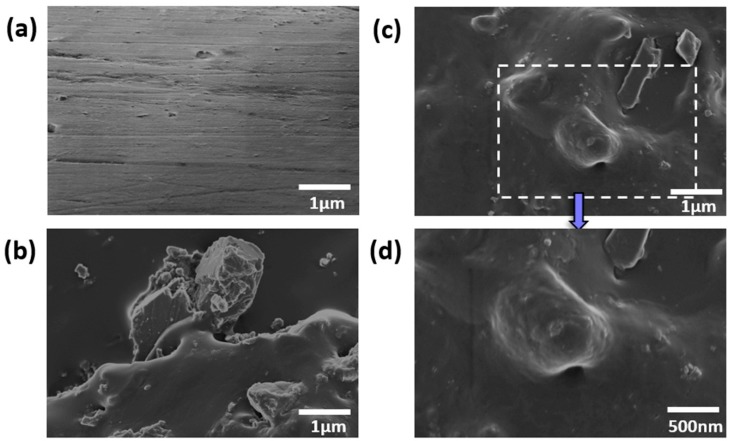
FE-SEM images of (**a**) the neat WSPU, (**b**) WSPU/20CaCO_3_, and (**c**) WSPU/20TCaCO_3_; (**d**) magnified image of WSPU/20TCaCO_3._

The morphologies of both the WSPU and WSPU composites were examined by FE-SEM in order to investigate the distribution of the CaCO_3_ particles and their adherence to the WSPU. The FE-SEM images of the WSPU and WSPU/CaCO_3_ composites are presented in Figure 7. Sufficiently good levels of adhesion and dispersion of the CaCO_3_ particles are essential for the filler to improve the final properties of the polymer composites. In Figure 7a, the nitrogen-fractured neat WSPU displays a smooth surface, while the composite (Figure 7b,c) displays particles distributed over the polymer surface. Moreover, a comparison Figure 7b,c indicates good adherence of the surface-modified CaCO_3_ particles (WSPU/20TCaCO_3,_ Figure 7c) whereas the high surface energy of the pristine CaCO_3_ particles led to their exposure on the surface of the WSPU (WSPU/20CaCO_3_, Figure 7b) due to the lack of surface functionalization [9]. Hence, the surface treatment of the CaCO_3_ particles using APTES resulted in good dispersion and good adherence to the surface of the WSPU, as shown in Figure 7b.

**Figure 8:**
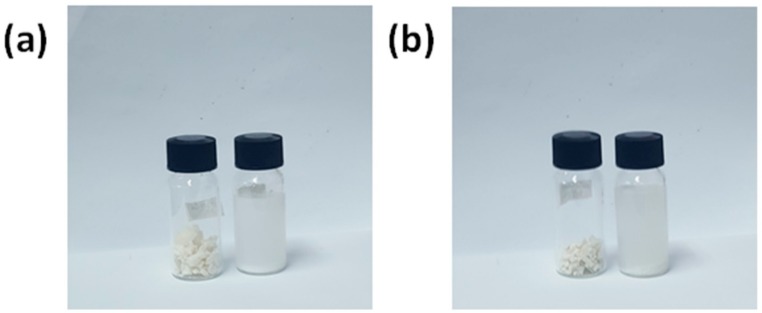
Water-solubility results: (**a**) WSPU/20CaCO_3_ and (**b**) WSPU/20TCaCO_3_.

The photographic images in Figure 8 present the water-solubility tests of the WSPU composites with (a) 10% and (b) 20% CaCO_3_ with surface modification. In each image, the right-hand vial shows the water-solubility results of the composite, while the left-hand vial shows the water solubility of the raw composite with modified CaCO_3_. The composites were soluble in water within three hours of stirring at room temperature. However, the addition of CaCO_3_ particles did not affect the water solubility of WSPU; rather, solubility was conferred by the emulsifier (DMPA).

**Figure 9:**
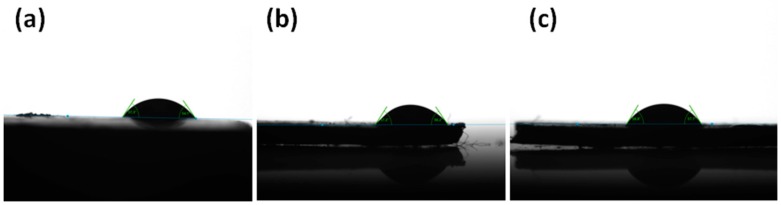
Contact angle of (**a**) neat WSPU, (**b**) WSPU/10TCaCO_3_ composite, and (**c**) WSPU/20TCaCO_3_ composite.

The contact angle measurements of the WSPU film and the flat surface of the WSPU/CaCO_3_ composite are presented in Figure 9. Due to the application of emulsifiers, both the neat WSPU and the WSPU/CaCO_3_ composites displayed hydrophilic properties and were thus water-soluble, as indicated by the above-mentioned water-solubility test. The application of CaCO_3_ did not affect the hydrophilic nature of the polymer, which may have been due to the immiscibility of the CaCO_3_ particles in water. The average contact angles were around 58°, thus confirming the water solubility of the composite. The images shown for neat WSPU are thicker than the composites, because it was a film that was not hot-pressed.

## 4. Conclusions

Water-soluble polyurethane (WSPU) composites were prepared from a soft segment of hydroxyl-terminated polybutadiene (HTPB) and polyethylene glycol (PEG, average molecular weight = 4000), and a hard segment of aliphatic diisocyanate (isophorone diisocyanate, IPDI) with dimethylolpropionic acid (DMPA) as emulsifier, triethyl amine (TEA) as a neutralizer, butane diol (BD) as a chain extender, and CaCO_3_ particles as a filler. The water solubility and hydrophobic nature were confirmed by water-solubility tests and contact angle measurements, and the composites were water-soluble and hydrophilic, respectively. The surface of the CaCO_3_ particles was modified by using aminopropyltriethoxysilane (APTES), and the results of Fourier-transform infrared spectroscopy (FTIR) confirmed the successful attachment of APTES to the surface of the CaCO_3_ particles. The structure of the WSPU was confirmed by FTIR, which indicated the formation of urethane bonds. The distribution of the CaCO_3_ particles in the WSPU matrix and their adhesion to the surface were confirmed by FE-SEM, which indicated that the surface-modified CaCO_3_ was well-distributed and displayed good adhesion. By contrast, the pristine CaCO_3_ was found to form aggregates in the matrix and did not adhere to the surface. The thermal degradation properties were determined by TGA analysis, which indicated the presence of various degradation regions and the improvement in the thermal degradation properties of WSPU with the addition CaCO_3_.
